# Assessing radiotracer kinetics in the Langendorff perfused heart

**DOI:** 10.1186/2191-219X-3-74

**Published:** 2013-11-14

**Authors:** Erika Mariotti, Mattia Veronese, Joel T Dunn, Rodolfo A Medina, Philip J Blower, Richard Southworth, Thomas R Eykyn

**Affiliations:** 1 Division of Imaging Sciences and Biomedical Engineering, St. Thomas' Hospital, King's College London, London SE1 7EH, UK; 2 Institute of Psychiatry, King's College London, London SE5 8AF, UK; 3 CRUK and EPSRC Cancer Imaging Centre, Royal Marsden NHS Trust and The Institute of Cancer Research, Sutton, Surrey SM2 5NG, UK

**Keywords:** PET, Spectral analysis, Kinetic modelling, [^18^ F]-FDG, [^18^ F]-FMISO, Perfused heart

## Abstract

**Background:**

The Langendorff perfused heart is a physiologically relevant and controllable model with potential for assessing the pharmacokinetics of new radiotracers under a range of pathophysiological conditions.. We assess the feasibility of extending the methods validated for in *vivo* PET data analysis to the characterisation of PET tracer kinetics applied to Langendorff perfused hearts.

**Methods:**

Monte Carlo simulations were used to study the accuracy and reproducibility of linear and non-linear spectral analysis (SA/NLSA), the Patlak graphical method and normalised tissue activity (NA). The methods were used to analyse time-activity curves of two widely used PET tracers, [^18^ F]-FDG and [^18^ F]-FMISO, acquired *ex vivo* from Langendorff perfused rat hearts under normoxic and hypoxic conditions.

**Results:**

Monte Carlo simulations showed NLSA to be superior to SA in identifying and quantifying the presence of irreversible trapping component (*α*_o_), for low values of *α*_o_. The performance of NLSA and SA for high values of trapping was comparable. NLSA was also more precise than SA in determining the absence of trapping over the range of simulated kinetics and SNR. Simulations also suggest that the semi-quantitative method NA is adequate for the evaluation of trapping, and it was found to be more accurate than Patlak. The values of *α*_0_ estimated with NLSA from the time series of both [^18^ F]-FDG and [^18^ F]-FMISO increased significantly from normoxia to hypoxia in agreement with previous studies. The values of trapping derived using SA increased but not significantly, reflecting the larger error associate with this method. Patlak estimated from the experimental datasets increased from normoxia to hypoxia but was not significant. NA estimated from the [^18^ F]-FDG data increased from normoxia to hypoxia, but was not significant, whilst NA calculated for [^18^ F]-FMISO time-activity curves increased significantly.

**Conclusions:**

Monte Carlo simulations suggested that spectral-based quantitative analysis methods are adequate for the kinetic characterisation of time-activity curves acquired *ex vivo* from perfused hearts. The uptake rate Patlak and the index NA also represent a good alternative to the SA and NLSA algorithms when the aim of the kinetic analysis is to measure changes in the amount of tracer trapped in the irreversible compartment in response to external stimuli. For low levels of trapping, NLSA and NA were subject to lower errors than SA and Patlak, respectively.

## Background

The isolated perfused heart is commonly used for the early evaluation of pharmaceuticals as well as being able to assess novel radiotracer pharmacokinetics [1-3]. It is a dynamic, biologically relevant model, with an intact vasculature, which provides insight into tracer uptake and retention in a metabolically active beating heart, without the potential complications of systemic metabolism and recirculation. It is versatile in terms of allowing direct drug and tracer administration; control of perfusion, workload and energy substrate supply; simple induction of hypoxia or ischemia; and the capacity to monitor the effects of such interventions upon cardiac contractile function and biochemistry [4-9].

**Figure 1:**
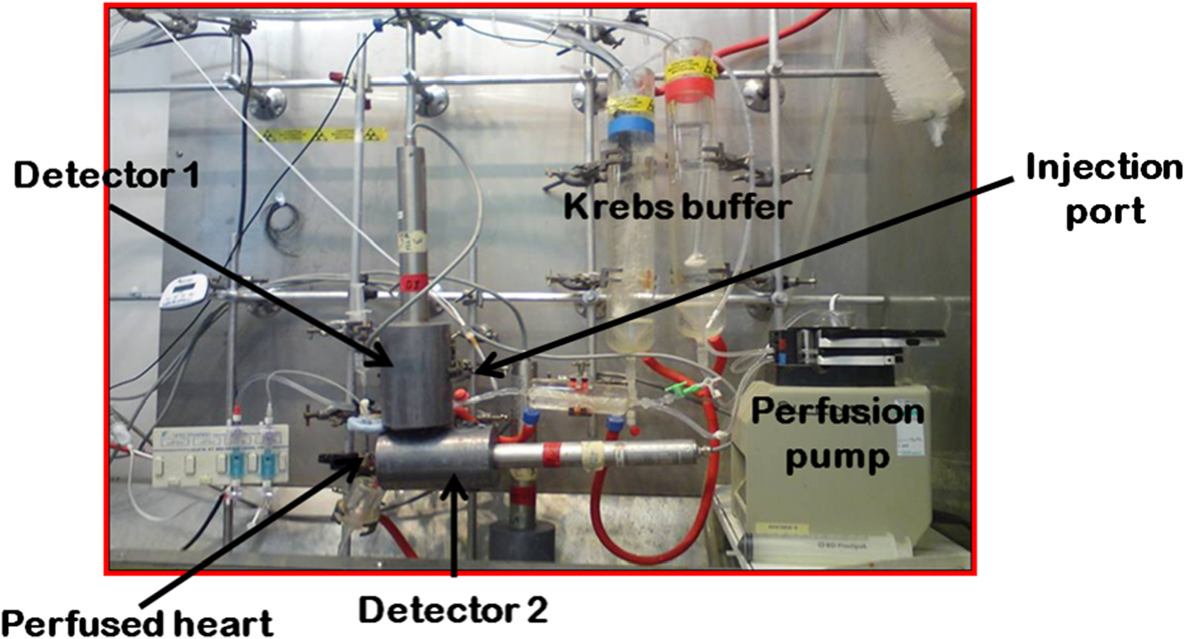
**Experimental set-up.** In each experiment, a bolus of [^18^ F]-FDG or [^18^ F]-FMISO was injected through the injection port, and its passage through the perfusion line was recorded using two NaI γ-detectors which are able to measure the input (detector 1) and the tissue (detector 2) time-activity curves.

*Ex vivo* time-activity curves of Positron Emission Tomography (PET) tracers from the isolated heart can be acquired through single or multiple NaI γ-detectors with no spatial information but very high temporal resolution [2,10]. Delivery input functions (*C*_in_(*t*)) can be measured in a Langendorff perfused heart with a γ-detector coupled to the inflow line, while time-activity curves in the heart (*C*_tiss_(*t*)) can be acquired using a single γ-detector interrogating the entire isolated perfused heart (see Figure 1). As tracer uptake is heterogeneous across the myocardium (from endocardium to epicardium, for example [11]), time-activity curves from perfused hearts likely represent a more heterogeneous range of kinetics than spatially resolved imaging data. *Ex vivo* time-activity curves further differ from those acquired *in vivo* in terms of signal-to-noise ratio (SNR) and lack of recirculation leading to an input function that decays rapidly to zero. In order to relate the measured activity to the underlying physiological or biochemical processes, the application of mathematical models to describe tracer kinetics is necessary.

A number of quantitative and semi-quantitative analysis methods are available for the evaluation of PET tracer kinetics *in vivo*, but to date, relatively few studies have investigated their validity for characterisation of PET tracers *ex vivo*, where there is the potential to gain biochemical insight into the nature of radiotracer trapping mechanisms or disease processes under more readily controlled experimental conditions. Previous studies have fitted time-activity curves from isolated hearts with exponential functions characterised by an arbitrary number of rates and quantified the tracer retention by averaging the activity measured in a chosen time interval at the end of the acquisition window [10]. This approach assumes the presence of an irreversible trapping component and the number of kinetic rates characteristic of a particular tracer *a priori*. The approach also rarely includes consideration of the delivery input function as a means of accurately quantifying tracer tissue kinetics.

In this study, we explore the possibility of extending the approaches used for the analysis of *in vivo* PET data to the kinetic analysis of time-activity curves acquired from Langendorff perfused rat hearts. We assess the accuracy and reproducibility of quantitative spectral analysis (SA) [12] and non-linear spectral analysis (NLSA) methods [13], as well as of the graphical method Patlak [14] and the semi-quantitative index normalised activity (NA) in both simulated and experimental *ex vivo* PET datasets. These methods were chosen because they can be applied to homogenous as well as heterogeneous systems without any *a priori* hypothesis on the number of compartments necessary to describe the data. Monte Carlo simulations were used to compare the performance of SA and NLSA in (1) identifying the presence of irreversible trapping, (2) deriving the number of kinetic components and (3) quantifying the irreversible trapping component from *ex vivo* time-activity curves. SA and NLSA were used to analyse experimental *ex vivo* time-activity curves acquired from isolated perfused rat hearts after the injection of two widely used PET tracers, [^18^ F]-FDG and [^18^ F]-FMISO, and compared with the uptake rate Patlak and the index NA in normoxic conditions and in response to hypoxia.

## Methods

### Spectral analysis

In SA, the tissue activity at time *t*, *C*_tiss_(*t*), is modelled as a convolution of the delivery input function, *C*_in_(*t*), with the sum of *M* + 1 distinct exponential terms as in Equation 1.

(1) $$C_{\mathrm{tiss}}\left(t\right)=\sum_{j=0}^{M}C_{\mathrm{in}}\left(t\right)⊗\alpha_{j}e^{-\beta_{j}t}$$

*α*_*j*_ and *β*_*j*_ are assumed to be real non-negative values. The upper limit, *M*, represents the maximum number of terms to be included in the model. The values of the rates *β*_*j*_ are predetermined and fixed in order to cover a range of all possible kinetic components measurable from the data. The values of the amplitudes *α*_*j*_ are estimated from the input and tissue time-activity curves by non-negative least square algorithm, and normally, only a few components with *α*_*j*_ > 0 are detected.

Spectral analysis was implemented as previously reported [15], with *C*_tiss_(*t*) referring to the activity of the tracer measured in the whole heart and *C*_in_(*t*) associated to the measured delivery input function. The grid of values of the rates *β*_*j*_ (*β*_1_ < *β*_2_ < … < *β*_*M*_) (Equation 1) was defined as a logarithmic distribution with lower limit *β*_1_ = 1/(3*T*_end_), where *T*_end_ was the end time of the experiment, and upper limit *β*_*M*_ = 3/*T*_in_ in agreement with previous studies [16]. *T*_in_ was the duration of the first time frame of the experiment. The number of points *M* between *β*_1_ and *β*_*M*_ was chosen equal to 100. A component for *β* = 0 was included in the model, corresponding to a fully trapped component. The *M* + 1 unknown values of *α*_*j*_ were estimated through a non-negative least square estimator. Weights were inversely proportional to the variance of decay-corrected measured activity. The SA algorithm was implemented in Matlab (the MathWorks, Natik, USA).

### Non-linear spectral analysis

For the implementation of NLSA, it is not necessary to specify a grid of values of *β*_*j*_. The *M* + 1 unknown values of *β*_*j*_ and *α*_*j*_ were estimated through non-linear fitting with the initial conditions chosen within a physiological range (and in agreement with [13]) maintained constant for all datasets analysed. The number of exponentials necessary to give a good fit of the data *M* (Equation 1) was fixed between 1 and up to a maximum of 4, in agreement with previous studies [13]. The Akaike information criterion (AIC) [17] was used to choose the model that best fit the data. Weights were inversely proportional to the variance of decay-corrected measured activity. The NLSA algorithm was implemented in Matlab (the MathWorks, Natik, USA).

### Graphical method: Patlak plot

Due to the demonstrated irreversible tissue retention of the tested tracers [13,18], the Patlak plot (given by the expression below) was chosen among the available graphical methods [14]:

(2) $$\frac{C_{\mathrm{tiss}}\left(t\right)}{C_{\mathrm{in}}\left(t\right)}=K\frac{\int_{0}^{t}C_{\mathrm{in}}\left(\tau \right)\mathrm{d\tau}}{C_{\mathrm{in}}\left(t\right)}+V$$

where *K* represents the net uptake rate of the tracer and *V* the distribution of the tracer in the compartment that is in rapid equilibrium with the plasma. The unknown constants *K* and *V* were obtained by linear regression from a graph of *C*_tiss_(*t*)/*C*_in_(*t*) against $\int_{0}^{t}C_{\mathrm{in}}\left(\tau \right)\mathrm{d\tau}/C_{\mathrm{in}}\left(t\right)$ computed for *t* > 7.5 min. Because of the large number of data points (five samples per second), both quantities were interpolated on a 6-s sub-sampled grid. *K* was the ultimate parameter of interest considered, while *V* was excluded from the tracer kinetic analysis.

### Normalised activity

We defined the NA as a surrogate of the semi-quantitative index standard uptake value (SUV) used for the characterisation of tracer trapping *in vivo*. SUV is given by the ratio of the tissue radioactivity and the injected radioactivity divided by the body weight [19]. In *ex vivo* experiments, the input function is delivered as an impulse with no recirculation, and therefore, the plasma radioactivity rapidly decays to zero. Additionally, due to the experimental set-up, the measured activity is not directly proportional to the radioactivity concentration. For these reasons, the semi-quantitative index SUV could not be computed in this study as it is defined for *in vivo* experiments. We define a new index, NA, in which the maximum measured activity in the target tissue substituted the normalised injected dose of the tracer normally used in SUV. NA was calculated as a ratio of the mean tissue activity (*C*_tiss_(*t*)) measured in counts per second (CPS) over a small interval at the end of the experiment [end - 0.075 min, *t*_end_] and the maximum value of *C*_tiss_(*t*) (CPS).

(3) $$\mathrm{NA}=\frac{\mathrm{mean}\langle C_{\mathrm{tiss}}\left(t_{\mathrm{end}-0.075\min},t_{\mathrm{end}}\right)\rangle }{\max\left(C_{\mathrm{tiss}}\left(t\right)\right)}$$

### Simulation studies

**Figure 2:**
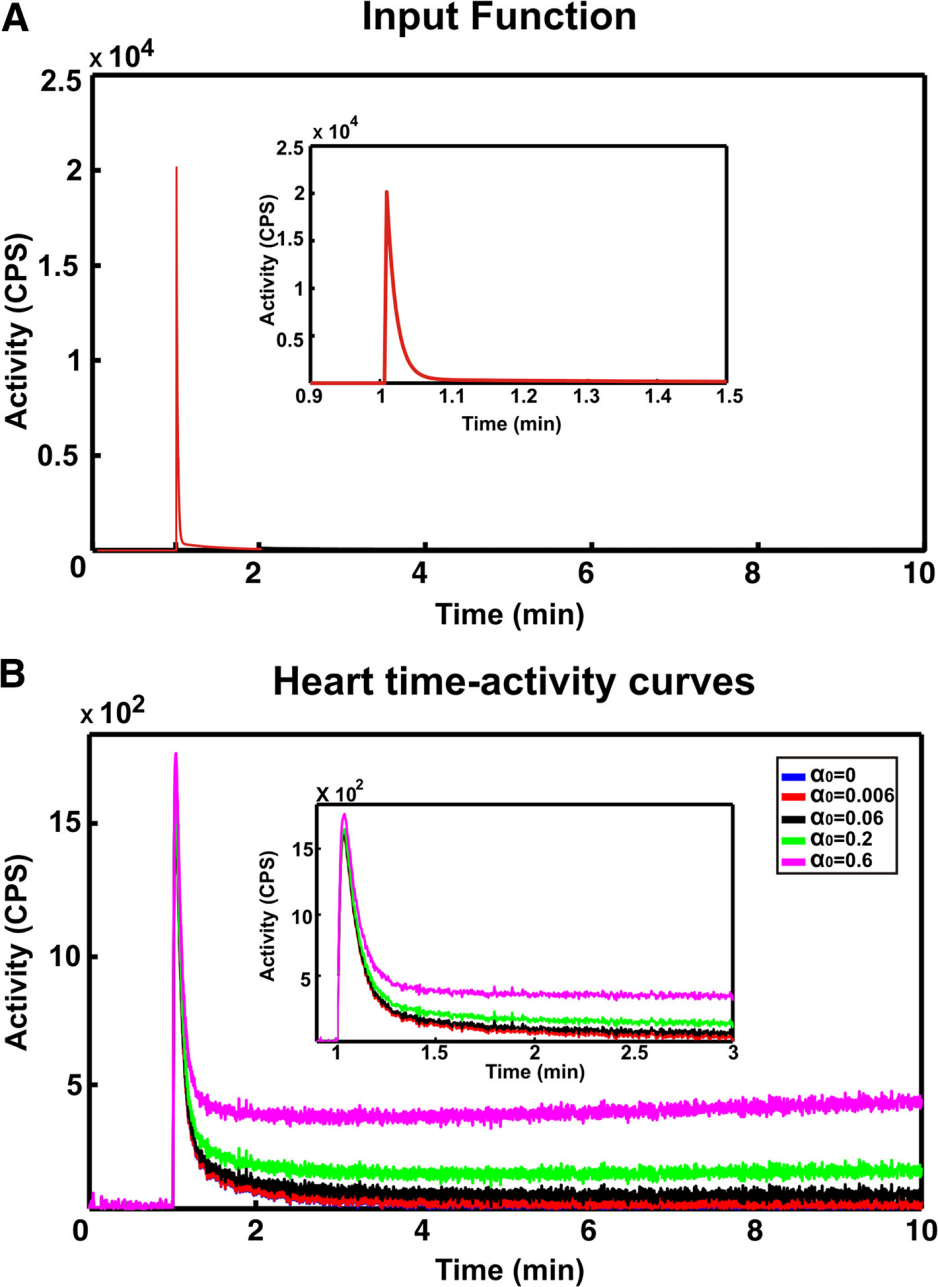
**Simulated datasets. (A)** Representative input function used in the simulations. **(B)** Representative simulated heart time-activity curves for different values of irreversible trapping components (*α*_0_). The dynamics simulated were chosen to mimic the tissue time-activity curves of hypoxia-sensitive tracers characterised by an increase in tracer uptake when the perfused heart is made hypoxic compared to that measure in normoxia.

Datasets were simulated, reproducing the characteristics of *ex vivo* time-activity curves from isolated hearts in normoxia and at different levels of hypoxia. Bi- and tri-exponential *ex vivo* time-activity curves with known kinetics were simulated using Equation 1. The values of *β*_*j*_ were fixed (for tri-exponentials, *β*_1_ = 0.5 min^-1^, *β*_2_ = 5 min^-1^ and *β*_3_ = 15 min^-1^; and for bi-exponentials, *β*_1_ = 0.5 min^-1^and *β*_2_ = 15 min^-1^), whereas *α*_*j*_ were randomly generated within a chosen interval (for tri-exponentials, 0.011 min^-1^ < *α*_1_ < 0.11 min^-1^, 0.12 min^-1^ < *α*_2_ < 0.2 min^-1^, and 5 min^-1^ < *α*_3_ < 8 min^-1^; and for bi-exponentials, 0.011 min^-1^ < *α*_1_ < 0.11 min^-1^ and 5 min^-1^ < *α*_2_ < 8 min^-1^). Bi- and tri-exponential curves were simulated with and without a trapping component *α*_0_ (for *β*_0_ = 0). Four different values of irreversible trapping were simulated (*α*_0,1_ = 0.006 min^-1^, *α*_0,2_ = 0.06 min^-1^, *α*_0,3_ = 0.2 min^-1^ and *α*_0,4_ = 0.6 min^-1^) (see Figure 2). The values used in the simulations were chosen within a physiological range and in agreement with previous studies [13]. To obviate further sources of variability between one dataset and another, the same *C*_in_(*t*) was taken from an experimental dataset, representative of these experiments, and used for all simulations. Each dataset was simulated for a low and a high SNR (SNR = 60 and SNR = 120), consistent with the experimental data, and *N* = 100 and a time resolution Δ*t* = 0.0033 min were chosen. SNR was computed as the ratio of the maximum value of the time-activity curve and the standard deviation of the signal in the last minute of each experiment [20].

The percent bias (%BIAS) of quantitative and semi-quantitative indices (*α*_0_, Patlak and NA) was calculated as a performance index (Equation 4).

(4) $$\%{\mathrm{BIAS}}_{p}=100\;\times \sum_{j=1}^{k}\frac{\left(p_{j}-p_{\mathrm{TRUE}}\right)}{p_{\mathrm{TRUE}}}$$

where *p*_*j*_ and *p*_TRUE_ are the estimated and true value of the indices *p*.

### Experimental protocol

All procedures were performed in accordance with the United Kingdom Home Office Guide on the Operation of the Animals (Scientific Procedures) Act 1986 and KCL's Ethical Review Process Committee.

[^18^ F]-FDG was provided by the clinical PET Centre, St. Thomas' Hospital, whereas [^18^ F]-FMISO was prepared following a previously reported method [21].

Mature male Wistar rats (250 to 300 g) were fed *ad libitum* with regular animal feed. Hearts (*n* = 4 for [^18^ F]-FDG and *n* = 3 for [^18^ F]-FMISO) were harvested under terminal anaesthesia (sodium pentobarbitone (100 mg/kg) intraperitoneal injection with heparin (200 IU)) and plunged into ice-cold Krebs Henseleit buffer (KHB), with the following composition: NaCl 118 mM, NaHCO_3_ 25 mM, MgSO_4_ 1.2 mM, KCl 5.9 mM, Na_2_EDTA 0.6 mM, glucose 11.1 mM and CaCl_2_ 2.5 mM, pH 7.4. Hearts were cannulated via the aorta and retrogradely perfused at constant flow (14 ml/min) with KHB at 37°C. Cardiac contractile function was monitored with a water-filled balloon inserted into the left ventricular lumen inflated to record an end diastolic pressure of 6 to 8 mmHg, which was connected to a pressure transducer and recording apparatus. Perfusion pressure was measured by a further pressure transducer inserted into the arterial line. A bolus of each radiotracer (1 MBq in 100 μl KHB) was administered via an in-line injection port, and its transit through the perfusion apparatus was monitored by NaI detectors (1) in the arterial line above the heart cannula and (2) directly opposite the heart (to quantify the tracer accumulation in the heart) connected to a GinaSTAR TM ITLC unit (see Figure 1). All datasets were acquired with a time resolution Δ*t* = 0.0033 min.

**Figure 3:**
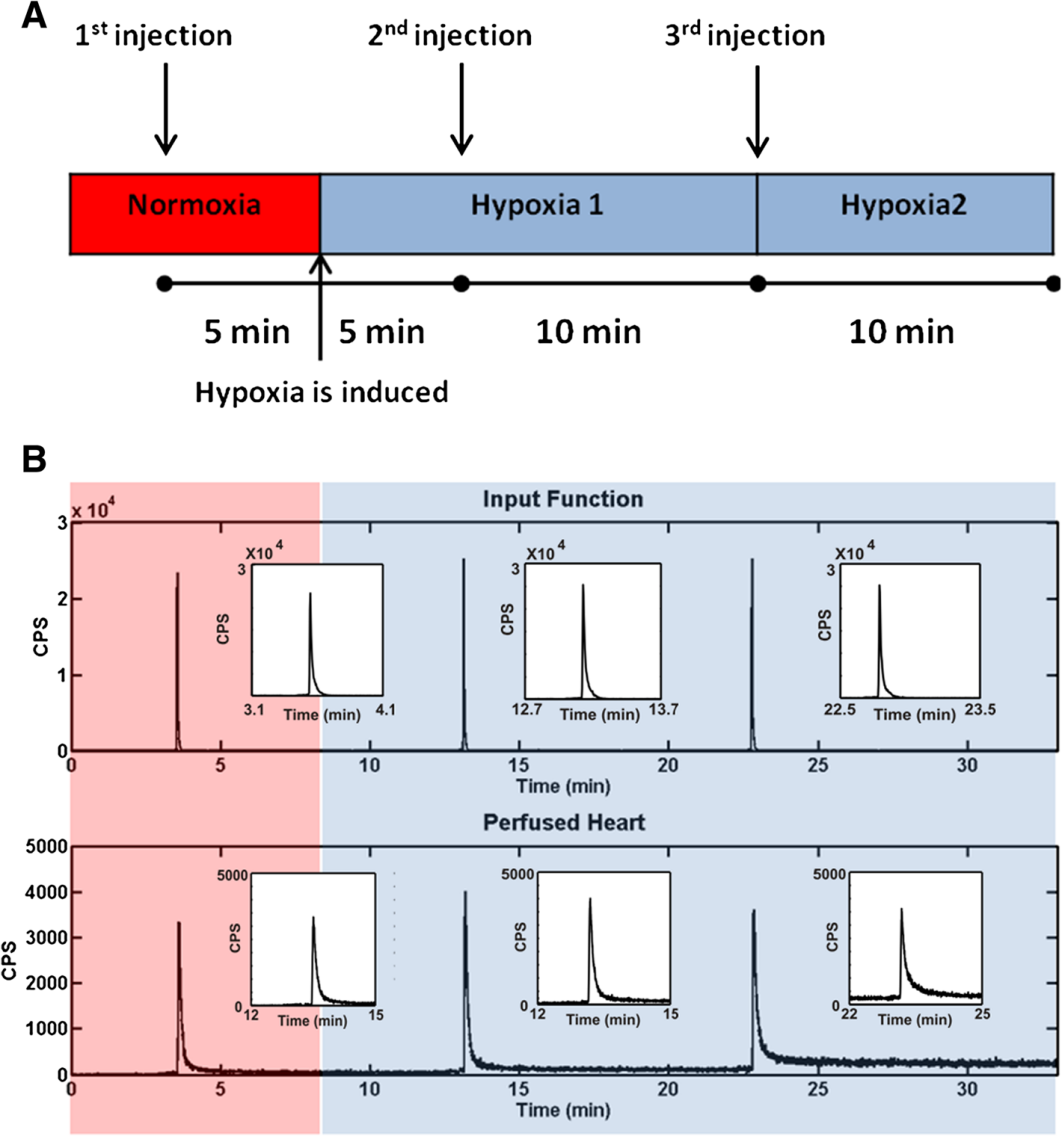
**Experimental protocol. (A)** Schematic representation of the experimental protocol used to acquire [^18^ F]-FDG and [^18^ F]-FMISO *ex vivo* time-activity curves from perfused rat hearts. After a stabilisation period, where the KHB perfusate was gassed with 95% O_2_-5% CO_2_, the first bolus of either [^18^ F]-FDG or [^18^ F]-FMISO was injected into the perfusion line. After 5 min, hypoxia was induced by switching to a second reservoir with KHB gassed with 95% N_2_-5% CO_2_. Further boli of each tracer were then injected into the system after 5 min and 15 min of hypoxic buffer perfusion, respectively. **(B)** Representative [^18^ F]-FDG input and tissue time-activity curves acquired *ex vivo* using the protocol described in (A) (*y*-axis units are counts per second).

After a stabilisation period, where KHB perfusate was gassed with 95% O_2_-5% CO_2_, the first bolus of either [^18^ F]-FDG or [^18^ F]-FMISO was injected into the perfusion line, and their passage through the system under 'normoxic’ conditions was recorded. After 5 min, hypoxia was induced by switching to a second reservoir (KHB gassed with 95% N_2_-5% CO_2_). Further boli of each tracer were injected into the system after 5 min and 15 min of hypoxic buffer perfusion, respectively, and the kinetics of cardiac retention monitored. A scheme of the experimental protocol used to acquire [^18^ F]-FDG and [^18^ F]-FMISO plasma and tissue time-activity curves is shown in Figure 3A, whereas a representative experimental dataset is shown in Figure 3B.

### Data correction

All datasets were corrected for radioactive decay. Additionally, to adjust the experimental data for residual activity due to prior injections in the same heart, a model prediction correction was used. The normoxic input/tissue time-activity curve was fitted with the algorithm assessed (SA or NLSA), and the result was extrapolated in the time interval relative to the second injection. The contribution of the activity from the first injection to the second was therefore calculated and subtracted. After the background correction, the curve relative to the second injection was fitted and its contribution subtracted to the third injection.

### Statistical analysis

Statistical analysis was performed using GraphPad Prism® (GraphPad Software Inc, USA). All values are expressed as the mean ± SD. Data were analysed using a one-way ANOVA with Dunnett's test to compare each group of datasets acquired in hypoxia with the corresponding control group measured in normoxia [22].

## Results

### Simulations

The results from the Monte Carlo simulations highlighted differences in performance of the two assessed spectral-based algorithms, the uptake rate Patlak and the semi-quantitative index NA.

Results from Monte Carlo simulations showed that NLSA is more accurate in identifying the absence of irreversible trapping (*α*_0_ = 0 min^-1^) for the dynamics and SNRs considered (see Table 1). For the lowest value of the trapping simulated (*α*_0_ = 0.006 min^-1^) with tri-exponential kinetics, a similar degree of accuracy was found for SA and NLSA, whereas for bi-exponential kinetics, SA was found to be superior. For higher values of trapping, the accuracy in estimating the presence of the trapping of the two algorithms was equal to 100% (see Table 1).

**Table 1:** **Proportion of simulations that had *α***_**0**_ **= 0 or *α***_**0**_ **≠ 0 identified by NLSA and SA**

**Simulated scenario**	**NLSA performance**						**SA performance**					
	**SNR = 60**			**SNR = 120**			**SNR = 60**			**SNR = 120**		
	***α***_**0**_ **= 0 (%)**	***α***_**0**_ **≠ 0 (%)**	**Accuracy (%)**	***α***_**0**_ **= 0 (%)**	***α***_**0**_ **≠ 0 (%)**	**Accuracy (%)**	***α***_**0**_ **= 0 (%)**	***α***_**0**_ **≠ 0 (%)**	**Accuracy (%)**	***α***_**0**_ **= 0 (%)**	***α***_**0**_ **≠ 0 (%)**	**Accuracy (%)**
Tri-exponential												
No trapping (*α*_0_ = 0)	100	-	100	100	-	100	21	79	21	14	86	14
Trapping 1 (*α*_0_ = 0.006)	10	90	90	6	94	94	6	94	94	10	90	90
Trapping 2 (*α*_0_ = 0.06)	-	100	100	-	100	100	-	100	100	-	100	100
Trapping 3 (*α*_0_ = 0.2)	-	100	100	-	100	100	-	100	100	-	100	100
Trapping 4 (*α*_0_ = 0.6)	-	100	100	-	100	100	-	100	100	-	100	100
Bi-exponential												
No trapping (*α*_0_ = 0)	100	-	100	100	-	100	14	86	14	2	98	2
Trapping 1 (*α*_0_ = 0.006)	23	77	77	13	87	87	4	96	96	-	100	100
Trapping 2 (*α*_0_ = 0.06)	6	94	94	2	98	98	-	100	100	-	100	100
Trapping 3 (*α*_0_ = 0.2)	-	100	100	-	100	100	-	100	100	-	100	100
Trapping 4 (*α*_0_ = 0.6)	-	100	100	-	100	100	-	100	100	-	100	100

*α*_0_ indicates the tracer trapping. Units are in min^-1^.

**Figure 4:**
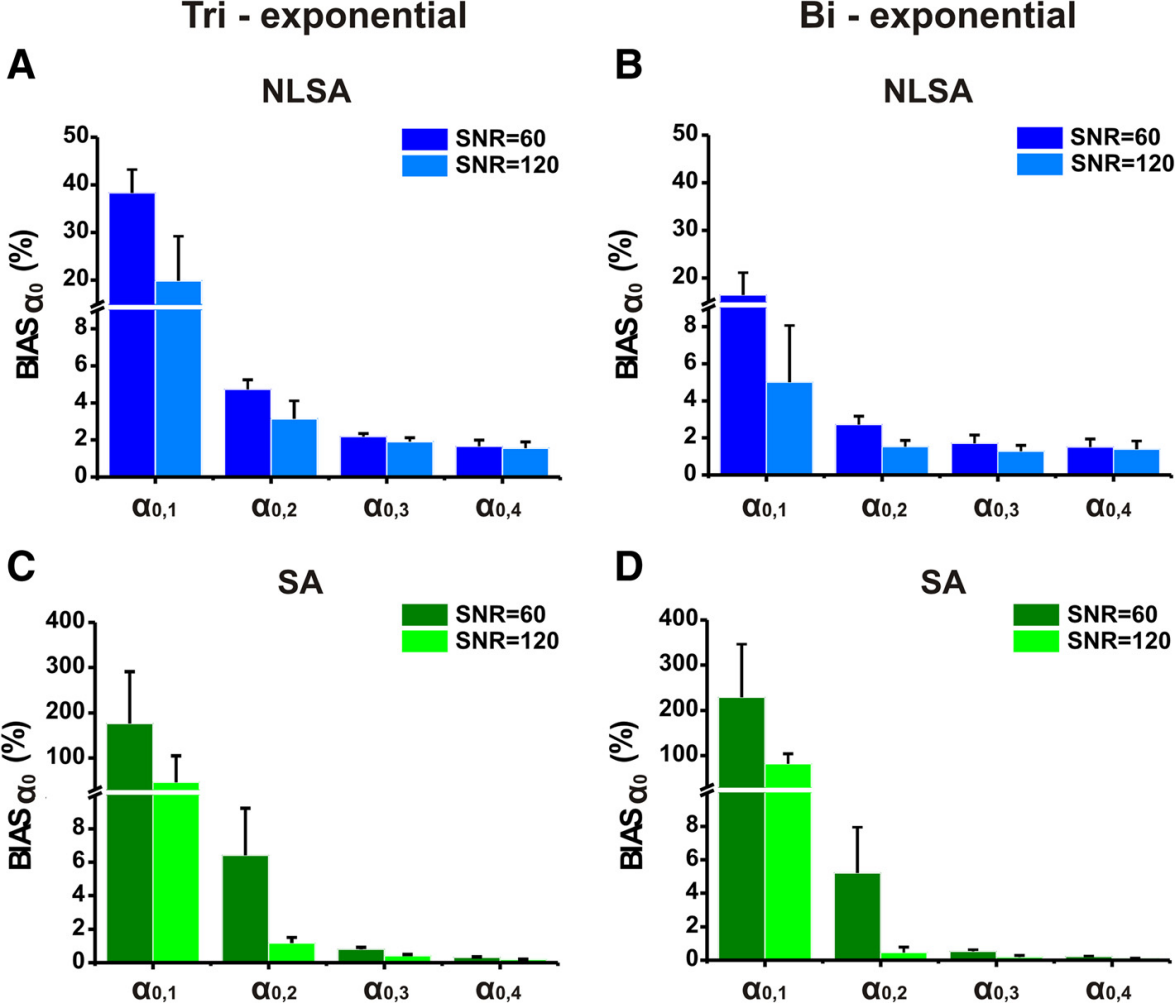
**Results for the estimation of *α***_**0 **_**from the Monte Carlo simulations.** Bias (percent) in calculated trapping (*α*_0_) of the NLSA and SA algorithm applied to simulated **(A, C)** tri-exponential and **(B, D)** bi-exponential models. *α*_0*,i*_ indicates the trapping of the tracer with the values reported in Table 1. Units are in min^-1^.

The mean and the standard deviation of the %BIAS*α*_0_ (Equation 4) reported with NLSA and SA for both bi- and tri-exponential curves and high and low SNRs are shown in Figure 4. For tri-exponential kinetics (see Figure 4A,C), NLSA displayed a lower bias than SA for the quantification of small *α*_0_ values with a maximum mean ± SD percentage bias equal to 38.30% ± 4.92% for NLSA compared to 176.15% ± 114.65% for SA at *α*_0,1_ = 0.006 min^-1^ and SNR = 60. For higher values of trapping (*α*_0,3_ = 0.2 min^-1^ and *α*_0,4_ = 0.6 min^-1^), SA and NLSA were of similar accuracy, with a minimum mean ± SD %BIAS*α*_0_ equal to 0.17% ± 0.03% for SA, compared to 1.55% ± 0.34% for NLSA at *α*_0,4_ = 0.6 min^-1^ and SNR = 120. The same trend was found for bi-exponential curves (see Figure 4B, D), where the maximum mean ± SD %BIAS*α*_0_ were 16.41% ± 4.71% for NLSA and 228.39% ± 117.70% for SA, at *α*_0,1_ = 0.006 min^-1^ and SNR = 60. The minimum mean ± SD %BIAS*α*_0_ values for high trapping were similar with those found for tri-exponential kinetics. The accuracy in quantification of *α*_0_ for both NLSA and SA algorithms improved with increasing values of trapping and SNR. Additionally, the mean ± SD percentage bias reported for both algorithms was generally lower for bi-exponential curves than those estimated for tri-exponential kinetics.

**Figure 5:**
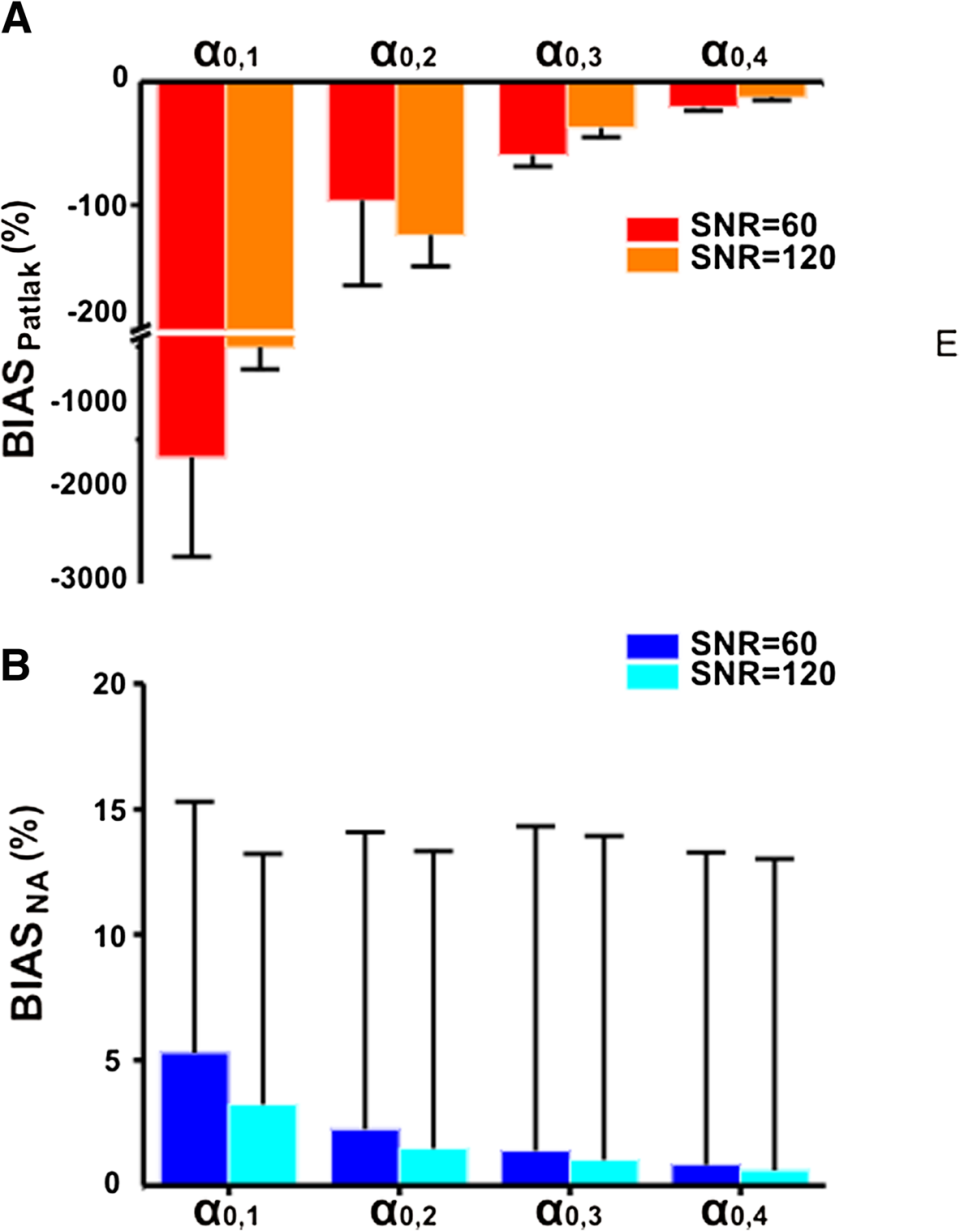
**Results for Patlak and NA from the Monte Carlo simulations.** Bias (percent) reported for **(A)** Patlak and **(B)** NA applied to simulated tri-exponential time-activity curves.

In Figure 5A,B, the mean and the standard deviation of the %BIAS reported for Patlak and NA are presented for simulated tri-exponential curves. In the absence of noise, Patlak returns the exact value of *α*_0_, whereas NA represents a surrogate of the trapping component which is in a linear relationship. The accuracy in quantifying the trapping component from the simulated data using Patlak increased with the value of trapping and SNR with a maximum mean ± SD percentage bias equal to -1,662% ± 1,053.8% at SNR = 60 and -507% ± 230% at SNR = 120 and a minimum mean ± SD percentage bias equal to -20.2% ± 3% at SNR = 60 and -12.2% ± 2.5% at SNR = 120. The maximum mean ± SD percentage bias reported for NA is 5.3% ± 10% (a.u.) at SNR = 60 and 3.2% ± 10% (a.u.) at SNR = 120, while the minimum mean ± SD percentage bias is equal to 0.8% ± 12.4% (a.u.) at SNR = 60 and 0.6% ± 12.4% (a.u.) at SNR = 120. The values reported for the %BIAS of both Patlak and NA estimated from bi-exponential curves were not significantly different from those calculated for tri-exponential curves (data not reported).

Table 2 shows the performance of the NLSA and SA algorithm in estimating the number of non-trapped components from simulated bi- and tri-exponential curves. Results showed the percentage of datasets where the number of components was under estimated (UE), over estimated (OE) or correctly estimated (CE). For tri-exponential time-activity curves, the accuracy in determining the number of components was better for NLSA when *α*_0_ = 0 min^-1^, whilst SA was better at all other values of *α*_0_ simulated. For bi-exponential kinetics, the accuracy in determining the number of components was significantly better than for tri-exponential time-activity curves. NLSA was more accurate when *α*_0_ = 0 min^-1^, whereas SA was more accurate than NLSA for all non-zero values of trapping simulated.

**Table 2:** Proportion of simulations that had the number of components underestimated, overestimated or correctly estimated by NLSA and SA

**Simulated scenario**	**NLSA performance**						**SA performance**					
	**SNR = 60**			**SNR = 120**			**SNR = 60**			**SNR = 120**		
	**UE (%)**	**OE (%)**	**CE (%)**	**UE (%)**	**OE (%)**	**CE (%)**	**UE (%)**	**OE (%)**	**CE (%)**	**UE (%)**	**OE (%)**	**CE (%)**
Tri-exponential												
No trapping (*α*_0_ = 0)	85	-	15	0	-	100	-	81^a^	19^a^	-	100^a^	0^a^
Trapping 1 (*α*_0_ = 0.006)	100	-	0	46	-	54	18	-	82	-	-	100
Trapping 2 (*α*_0_ = 0.06)	100	-	0	60	-	40	18	-	82	-	-	100
Trapping 3 (*α*_0_ = 0.2)	100	-	0	95	-	5	46	-	54	-	-	100
Trapping 4 (*α*_0_ = 0.6)	100	-	0	100	-	0	53	-	47	1	-	99
Bi-exponential												
No trapping (*α*_0_ = 0)	-	-	100	-	-	100	-	86^a^	14^a^	-	-	100^a^
Trapping 1 (*α*_0_ = 0.006)	1	-	99	-	-	100	17	18	65	-	-	100
Trapping 2 (*α*_0_ = 0.06)	1	-	99	-	-	100	-	-	100	-	-	100
Trapping 3 (*α*_0_ = 0.2)	11	-	89	5	-	95	-	-	100	-	-	100
Trapping 4 (*α*_0_ = 0.6)	34	-	66	32	-	68	-	-	100	-	-	100

*α*_0_ indicates the tracer trapping. Units are in min^-1^; UE, underestimated number of components; OE, overestimated number of components; CE, correctly estimated. Results are shown only for the percentage of datasets where the presence of baseline was correctly estimated (Table 1). ^a^An accuracy in baseline identification was <25%.

### Experimental datasets

Figure 3B shows representative time-activity curves acquired from an isolated perfused rat heart using the experimental set-up and protocol presented in Figures 1 and 3A, respectively. Cardiac contractile function of the perfused hearts was monitored throughout the experiment. The left ventricular end diastolic pressure (LVEDP) increased from 7 mmHg ± 2 mmHg in normoxia to 29 mmHg ± 6 mmHg after 5-min hypoxia and further to 79 mmHg ± 8 mmHg after 15-min hypoxia. The left ventricular developed pressure (LVDP) decreased from 142 mmHg ± 5 mmHg in normoxia to 48 mmHg ± 5 mmHg after 5-min hypoxia and further to 25 mmHg ± 4 mmHg after 15-min hypoxia.

**Figure 6:**
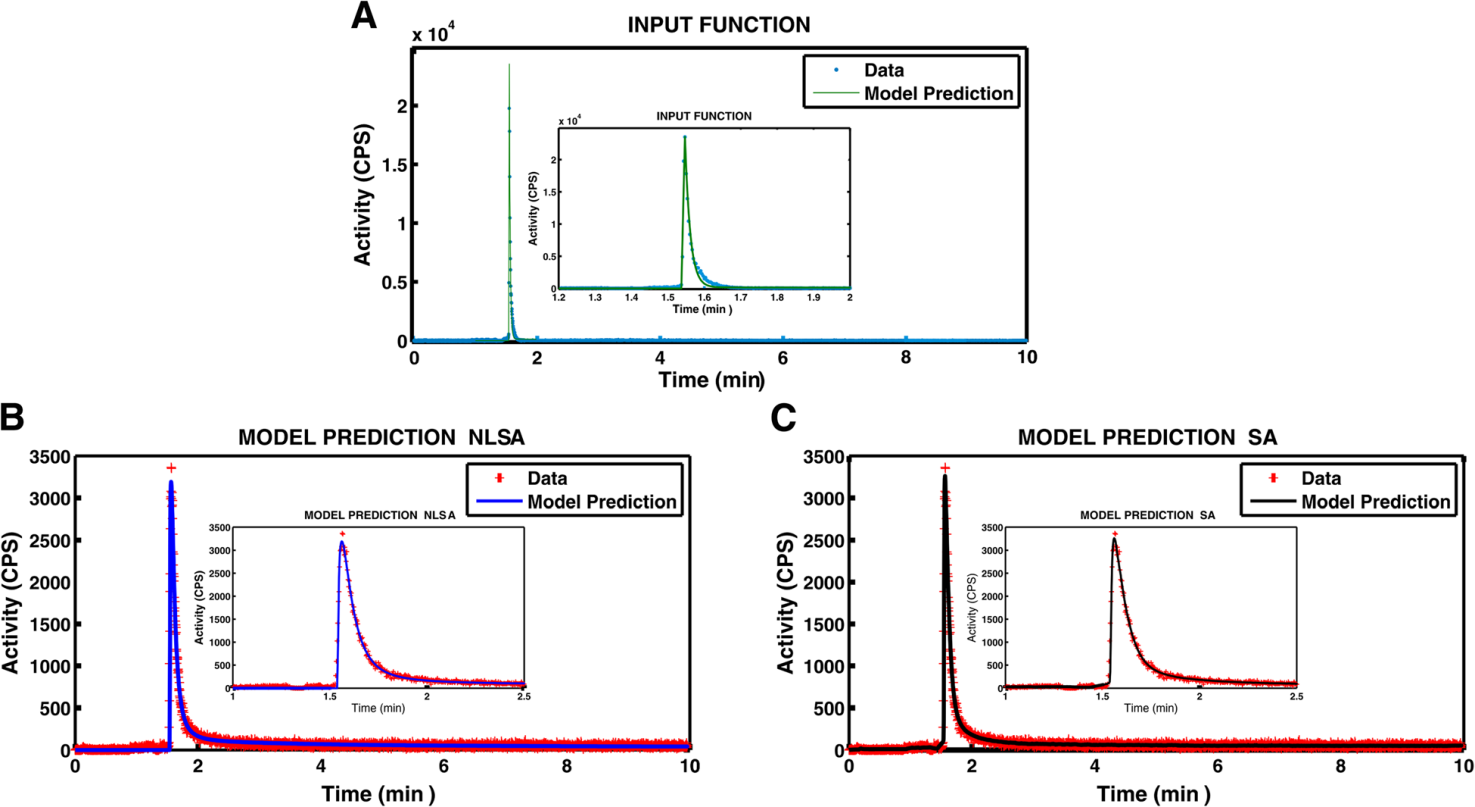
**Fitting of a representative [**^**18**^ **F]-FDG *ex vivo *dataset. (A)** Experimental input function (blue dots) with overlaid fit (green). **(B, C)** Experimental heart data (red dots) with overlaid NLSA (blue) and SA (black) fits.

Two time-activity curves were detected for each experiment: one representative of the delivery input function and a second reflecting the tracer accumulation in the heart. Both curves were corrected for the radioactive decay and residual tracer content in the heart from previous injections. In Figure 6, a representative normoxic dataset after decay and background correction (coloured dots) is shown with overlaid fits (coloured lines). The SNRs estimated for the [^18^ F]-FDG time-activity curves were 165 ± 40 in normoxia, 129 ± 35 at 5-min hypoxia and 103 ± 32 at 15-min hypoxia. The SNRs estimated for the [^18^ F]-FMISO time-activity curves were 253 ± 117 in normoxia, 182 ± 20 at 5-min hypoxia and 151 ± 17 at 15-min hypoxia.

Results from the NLSA and SA quantification of [^18^ F]-FDG datasets (*n* = 4) are shown in Table 3. The NLSA algorithm always identified a trapping component *α*_0_ (with the exception of one dataset run 4, hypoxia 1), whereas SA determined a null trapping component for 5 datasets out of 12. NLSA identified four kinetic components different from *α*_0_, while SA identified between three and five components.

**Table 3:** **Results from the NLSA and SA quantification for [**^**18**^ **F]-FDG *ex vivo *** **datasets**

	^**18**^ **F-FDG datasets**											
	**Normoxia**				**Hypoxia 1**				**Hypoxia 2**			
	**1**	**2**	**3**	**4**	**1**	**2**	**3**	**4**	**1**	**2**	**3**	**4**
*α*_0_ NLSA (min^-1^)	0.037	0.070	0.023	0.025	0.084	0.160	0.200	-	0.310	0.260	0.220	0.042
*α*_0_ SA (min^-1^)	0.038	0.005	0.037	-	0.064	-	-	-	-	0.130	0.050	0.060
Patlak (min^-1^)	0.066	0.080	0.066	0.040	0.100	0.150	0.250	0.046	0.200	0.180	0.300	0.077
NA (a.u.)	0.015	0.025	0.025	0.015	0.023	0.050	0.020	0.020	0.040	0.040	0.072	0.021

*α*_0_ indicates the tracer trapping. Numbers 1, 2, 3, and 4 indicate the run number.

**Figure 7:**
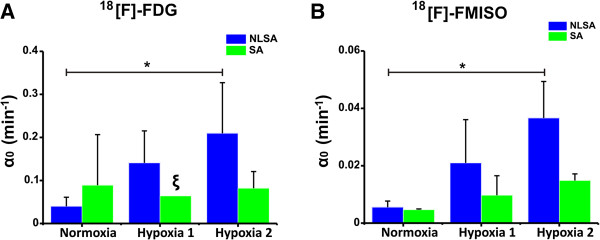
**Results from the NLSA and SA analysis of experimental datasets.** Mean ± SD of the trapping estimated with the NLSA (blue) and SA (green) algorithm in normoxia and hypoxia for **(A)** [^18^ F]-FDG and **(B)** [^18^ F]-FMISO experimental datasets. ξ indicates *n* = 1 and *p < 0.05. *α*_0_ indicates the trapping of the tracer.

**Figure 8:**
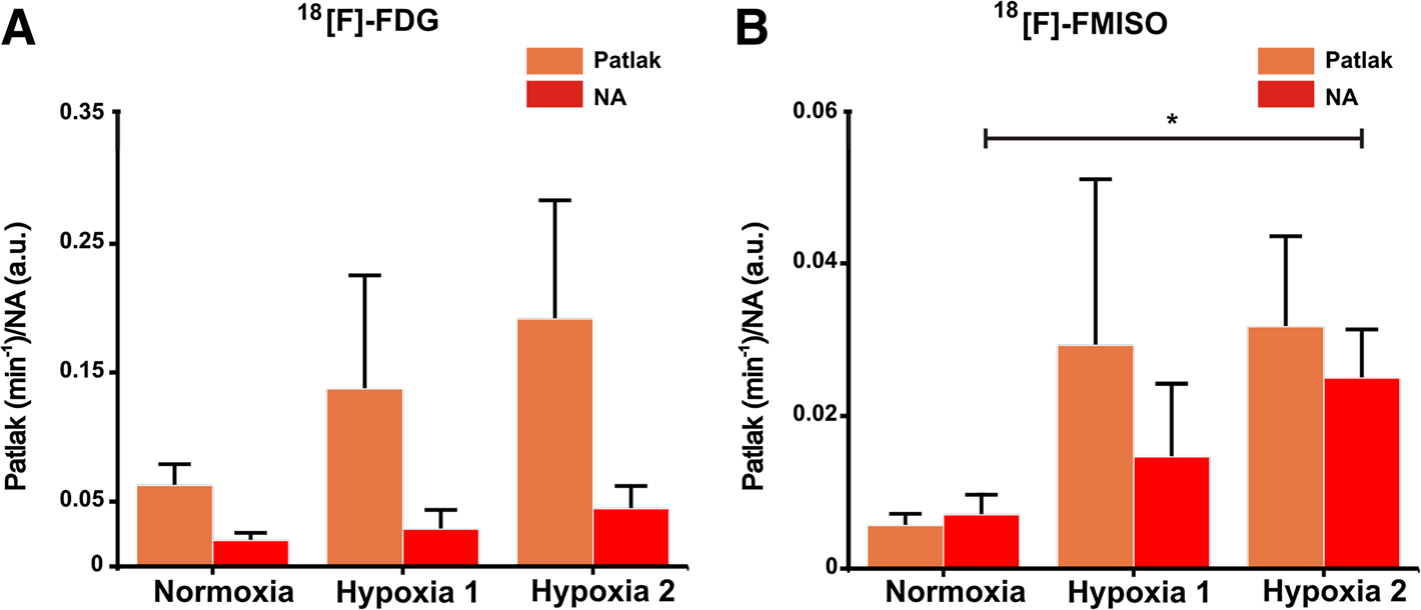
**Calculated values of Patlak and NA for experimental datasets.** Mean ± SD of Patlak (orange) and NA (red) in normoxia and hypoxia for **(A)** [^18^ F]-FDG and **(B)** [^18^ F]-FMISO experimental time-activity curve acquired *ex vivo* from perfused rat hearts. *p < 0.05.

Figure 7A shows the mean ± SD of the trapping quantified with NLSA and SA. The value of *α*_0_ estimated with NLSA significantly increased from 0.04 min^-1^ ± 0.02 min^-1^ in normoxia to 0.20 min^-1^ ± 0.11 min^-1^ at 15-min hypoxia (*p* < 0.05). The mean ± SD of the trapped component estimated with SA did not change significantly between normoxia and hypoxia and was associated with a larger error. The coefficient of variation (CV*α*_0_ (%)) associated with the estimation of the trapping component was smaller for NLSA than SA. The CV*α*_0_ (%) in normoxia was 3.4% ± 2% for NLSA and 181.2% ± 308.9% for SA. At 15-min hypoxia, it was 8.2% ± 7% and 36.3% ± 41.8% for NLSA and SA, respectively. Figure 8A shows the mean ± SD of Patlak and NA calculated for [^18^ F]-FDG. The value of Patlak increased, but not significantly, from 0.06 min^-1^ ± 0.02 min^-1^ in normoxia to 0.19 min^-1^ ± 0.09 min^-1^ at 15-min hypoxia. NA also increased from 0.03 ± 0.01 (a.u.) in normoxia to 0.04 ± 0.02 (a.u.) at 15-min hypoxia, but not significantly.

Table 4 shows the results from the NLSA and SA quantification for [^18^ F]-FMISO datasets (*n* = 3). The NLSA algorithm always identified a trapping component *α*_0_, whereas SA determined a null trapping component for one of the dataset (run 2, normoxia). The number of kinetic components different from *α*_0_ identified is four using NLSA and between two and four with SA. Figure 7B shows the mean ± SD of the trapping quantified with NLSA and SA. The value of *α*_0_ estimated with NLSA significantly increased from 0.005 min^-1^ ± 0.002 min^-1^ in normoxia to 0.03 min^-1^ ± 0.0002 min^-1^ at 15-min hypoxia (*p* < 0.05). The mean ± SD of the trapping component derived through SA also changed from 0.004 ± 0.00025 min^-1^ in normoxia to 0.0148 ± 0.0023 min^-1^ at 15-min hypoxia, but not significantly. As found for the [^18^ F]-FDG time-activity curves, the CV*α*_0_ (%) reported for NLSA was much smaller than that reported for SA. The CV*α*_0_ in normoxia was 4% ± 0.6% for NLSA and 16.1% ± 18.3% for SA. At 15-min hypoxia, it was 1.3% ± 0.2% and 13.7% ± 7% for NLSA and SA, respectively.

**Table 4:** **Results from the NLSA and SA quantification for [**^**18**^ **F]-FMISO *ex vivo *** **datasets**

	^**18**^ **F-FMISO datasets**								
	**Normoxia**			**Hypoxia 1**			**Hypoxia 2**		
	**1**	**2**	**3**	**1**	**2**	**3**	**1**	**2**	**3**
*α*_0_ NLSA (min^-1^)	0.004	0.008	0.004	0.033	0.025	0.011	0.050	0.040	0.023
*α*_0_ SA (min^-1^)	0.004	-	0.005	0.010	0.016	0.002	0.020	0.015	0.013
Patlak (min^-1^)	0.005	0.007	0.004	0.015	0.018	0.054	0.450	0.025	0.024
NA (a.u.)	0.004	0.007	0.010	0.020	0.020	0.004	0.028	0.028	0.017

*α*_0_ indicates the tracer trapping. Numbers 1, 2, 3, and 4 indicate the run number.

In Figure 8B, the mean ± SD of Patlak and NA calculated for [^18^ F]-FMISO time-activity curves is reported. The value of Patlak increased from 0.005 min^-1^ ± 0.001 min^-1^ in normoxia to 0.031 min^-1^ ± 0.01 min^-1^ at 15-min hypoxia, but was not significant. NA increased significantly from 0.007 ± 0.002 (a.u.) in normoxia to 0.02 ± 0.006 (a.u.) at 15-min hypoxia (*p* < 0.05).

## Discussion

The results from the Monte Carlo simulations suggest that both quantitative and semi-quantitative analysis methods are adequate for the kinetic characterisation of time-activity curves acquired *ex vivo* from perfused hearts. NLSA was found to be more accurate than SA in estimating the absence of trapping (*α*_0_ = 0 min^-1^) for the SNRs considered and for both tri- and bi-exponential kinetics. NLSA was also superior to SA in quantifying the trapping component at low values of *α*_0_, while for higher values of trapping, the performance of the two algorithms was comparable.

The difference in performance of NLSA and SA in the identification and quantification of the trapping component is due to the different implementation of the two algorithms. In SA, the possible values of the exponents *β*_*j*_ (Equation 1) that best describe the experimental data are chosen from a fixed grid of values and only the values of *α*_*j*_ are estimated. The optimal number of exponents is therefore not chosen *a priori* and is given by the number of rates *β*_*j*_ for which a non-null *α*_*j*_ is estimated. The presence of noise can significantly reduce the accuracy of SA in identifying and quantifying very low frequency (e.g. trapping) and high frequency components [15] as well as introducing phantom kinetic components [12,16]. For the NLSA algorithm, the experimental data are fitted with exponential curves of increasing order, and both *β*_*j*_ and *α*_*j*_ are derived using a non-linear least square estimator. In this case, all the kinetic components, specified *a priori*, are estimated, and the model that best fits the experimental data is chosen *a posteriori* using a standard model parsimony criterion [13]. This means that the very low frequency and high frequency components are always estimated and that the presence of noise can only affect the quantification of the value of the components, but not their identification.

SA was more accurate than NLSA in quantifying the number of components different from *α*_0_, regardless the value of the trapping and SNR for tri-exponential curves. To determine whether estimating the correct number of kinetic components using the NLSA algorithm was dependent upon on the standard model parsimony criterion used, we also implemented the SA algorithm where the optimal model was chosen using the Bayesian information criterion (BIC) rather than AIC. The AIC or BIC for a model is usually written in the form [-2log*L* + *kP*], where *L* is the likelihood function, *P* is the number of unknown parameters in the model and *k* is 2 for AIC and log(*n*) for BIC, with *n* number of points in the dataset. BIC therefore penalises a model characterised by a larger number of unknown parameters more than AIC does. However, using BIC as parsimony criterion rather than AIC, did not substantially improve the accuracy of the NLSA algorithm in estimating the number of kinetic components different from trapping.

The accuracy of both algorithms in determining the correct number of exponentials was significantly better for bi-exponential dynamics, with accuracy close to 100% for all cases studied. Two or more kinetic components with close values are likely to be seen by SA and NLSA as a unique component. The greater the spread of kinetic values, the easier it is for the algorithms to discern the exponentials as separate components. In our simulations, the values of the kinetic components *β*_*j*_ were more spaced in the bi-exponential than tri-exponential curves, meaning that it was easier for SA and NLSA to discern the two components as distinct *β*_*s*_.

The values estimated for Patlak and NA were found to be linearly proportional to the simulated values of trapping, but like spectral analysis, their estimation was affected by the presence of the noise. The bias (percent) associated with the estimation of Patlak was always higher than that reported for the semi-quantitative index NA, for all SNRs and trapping considered. Patlak is derived by calculating the ratio between tissue retention of the tracer and the delivery input function which decays rapidly to zero. Both curves exhibit noise, and therefore, dividing two noisy quantities amplifies the noise and reduces the accuracy of the method. The mean of the bias (percent) reported for NA becomes smaller with increasing values of trapping and SNRs, but the standard deviation does not change. In our Monte Carlo simulations for a given SNR, we added the same amount of noise to the time-activity curves with different values of the trapping component. The standard deviation of the bias is dependent on the noise variance of the time-activity curve fitted, and therefore, it does not change with increasing values of trapping for a given SNR.

For experimental datasets, the values reported for CV*α*_0_ (%) suggest that NLSA is more accurate than SA for the kinetic characterisation on *ex vivo* time-activity curves. NLSA identified a trapping component for the [^18^ F]-FDG datasets 11 times out of 12 and consistently indicated the presence of trapping for the [^18^ F]-FMISO datasets. Additionally, the trapping component quantified using NLSA significantly increases with the level of hypoxia for both [^18^ F]-FDG and [^18^ F]-FMISO, in agreement with previous studies [23]. In contrast, the Patlak values estimated from the experimental time-activity curves of [^18^ F]-FDG and [^18^ F]-FMISO (see Figure 8) are characterised by a large variability due to the noise exhibited by both tissue and input curves. An additional source of variability is associated with the nature of the *ex vivo* experiment where there is no recirculation. Bolus injection of radiotracer leads to a plasma activity that decays rapidly to zero, leading to an input function *C*_in_(*t*) mainly characterised by low SNR at later times.

*Ex vivo* time-activity curves have to be corrected for the contribution of radioactivity from previous injections to the residual tissue background signal in successive injections. For both spectral-based algorithms, we corrected for this residual activity by fitting the previous injection and then subtracting the contribution of the first injection to the second, and the contribution of the first and second to the third. This approach assumes that (1) the tracer studied is irreversibly trapped or, if reversible, has decayed entirely before the subsequent injection; (2) the concentration of exchangeable (reversible) tracer in the tissue is negligible before the next injection; and (3) the trapped component in a previous injection remains trapped when the condition of the heart is changed. With the tracers (irreversible) used in this study and the timing of their injections relative to their washout kinetics, the tracer activity in the heart contributing to each subsequent time-activity curve was very small and was mainly represented by trapped tracer with the activity of the tracer in the equilibrating compartments almost zero (<3% of the total measured signal). These conditions are necessary to guarantee that the trapping rates quantified after the first injection are associated with changes in tissue metabolism rather than the presence of residual activity in the tissue.

Monte Carlo simulations showed that both NLSA and SA are not able to reliably estimate the number of non-trapping components characterising the tracer kinetics especially when the number of rates is high. As a result, the kinetic analysis of time-activity acquired *ex vivo* from perfused hearts gives reliable information regarding the trapping component of the tracer studied, but no information of its transport/diffusion through the capillary membrane or of its transport from the intracellular space into the cells can be gained.

The Langendorff perfused heart has been the model of choice of cardiologist for years and has greatly increased our understanding of cardiac physiology. Nevertheless, this experimental model has a number of limitations. *Ex vivo* tissue time-activity curves are, for example, a heterogeneous measure of the tracer kinetics in the whole heart, as opposed to data acquired from a more spatially homogeneous voxel in imaging studies. It is expected that the number of kinetic components returned by SA approaches is higher than found within a given region of tissue [13] due to heterogeneity of tracer kinetics. While *in vivo* the coronary flow through the rat heart is 3 ml/min, in the buffer-perfused Langendorff heart, this increases to approximately 14 ml/min at the same perfusion pressure, due to the lower oxygen-carrying capacity of Krebs buffer. Because the flow rate defines tracer delivery to the tissue, this might complicate interpretation of semi-quantitative results where the bolus input is not accounted for. However, semi-quantitative approaches are simple to apply, and whilst less informative and more sensitive to experimental conditions, the use of NA offers a useful surrogate descriptor of tracer retention when datasets are acquired under an identical experimental set-up and protocol.

When investigating the pharmacokinetics of metabolic tracers like [^18^ F]-FDG, buffer substrate composition is also a critical consideration. In the present study, hearts were perfused with glucose as a sole energy substrate. The lack of fatty acids in the perfusion medium and their influence on glucose transport and or downstream glycolysis mean that [^18^ F]-FDG uptake may be different than that seen *in vivo*. More elaborate perfusion media and the use of membrane oxygenators would be required to study [^18^ F]-FDG metabolism under more physiologically appropriate conditions.

## Conclusions

Accurate quantification of radiotracer kinetics in the Langendorff perfused heart is highly desirable for the characterisation and development of novel radiotracers, and the exploitation of existing tracers to probe biological processes in greater detail. In this work, we compared the performance of three quantitative (SA, NLSA and Patlak) and one semi-quantitative (NA) analysis methods for the kinetic characterisation of *ex vivo* perfused heart time-activity curves. We tested the SA and NLSA algorithms in terms of the accuracy in identifying and quantifying the trapping component and in estimating the kinetics of non-trapping components. Results from both Monte Carlo simulations and experimental data suggest that NLSA is the algorithm of choice when the aim of the kinetic analysis is to assess whether the radiotracer injected in the perfused heart is irreversibly trapped or not. Once the presence of a fully trapped component has been identified, it can be quantified using either NLSA or the semi-quantitative index NA. Neither analysis methods were adequate for estimating the number of non-trapping components characterising the kinetics of *ex vivo* time-activity curves probably due to the heterogeneity of the experimental preparation.

## Competing interests

The authors declare that they have no competing interests.

## Authors’ contributions

EM implemented the analysis methods, analysed the data, participated in the design of the study and conceived and drafted the manuscript. MV participated in the implementation of the analysis methods and in the analysis data, as well as in the manuscript writing. JTD contributed to the design of the study and the interpretation of the data, as well as in the review of the manuscript. RAM and RS designed and carried out the perfusion experiments and participated in the critical review of the manuscript. PJB was involved in the interpretation of the data and in the manuscript review. TRE designed the study and was involved in the data analysis as well as in the review of the manuscript. All authors read and approved the final manuscript.
